# A rare case of invasive endometriosis causing intestinal obstruction

**DOI:** 10.1093/jscr/rjae082

**Published:** 2024-02-21

**Authors:** Sathish K Thirumurthy, Mahsheena Mohammed

**Affiliations:** Department of General Surgery, NMC Royal Hospital, Al Ghuwair, Sharjah, United Arab Emirates; Department of General Surgery, NMC Royal Hospital, Al Ghuwair, Sharjah, United Arab Emirates

**Keywords:** invasive endometriosis, intestinal obstruction, ileal endometriosis

## Abstract

A 35-year-old woman presented to the emergency department with severe right iliac fossa pain with features of subacute intestinal obstruction and recurrent episodes of similar pain in the past. CT scan showed a mass with fluid collection with no trace of the appendix in the right iliac fossa. The patient was taken up for a diagnostic laparoscopy and proceeded. Operative findings were that of a mass in the lumen of the terminal ileum just 6 inches from the ileocaecal junction. Normal pelvis with normal uterus and ovaries. The patient underwent a laparoscopic resection of the terminal ileum and limited resection of the ascending colon with an ileo-colic anastomosis. The patient recovered well and was discharged. The biopsy was reported as invasive endometriosis involving the muscularis layer of the terminal ileum with stricture of the terminal ileum with transmural inflammation. The case is being presented for the rarity of invasive endometriosis causing bowel obstruction with a normal pelvis.

## Introduction

Incidence of invasive endometriosis is a rare entity. Endometriosis usually occurs in menstruating women up to 15% [1]. Most common gastrointestinal involvement of endometriosis is found in the sigmoid colon, rectum, and terminal ileum in 3%–37% of women [2]. Proliferation and infiltration of the intestinal wall with endometrial implants may cause fibrotic reaction with formation of strictures and adhesions, probably from the effect of cyclical hormonal influences of menstruation. Eventually, this may lead to bowel obstruction and recurrent abdominal pain [3]. We are presenting an interesting case of invasive terminal ileum endometriosis required surgical resection.

## Methods

This is a case reported in line with SCARE criteria [4].

## Case presentation

**Figure 1:**
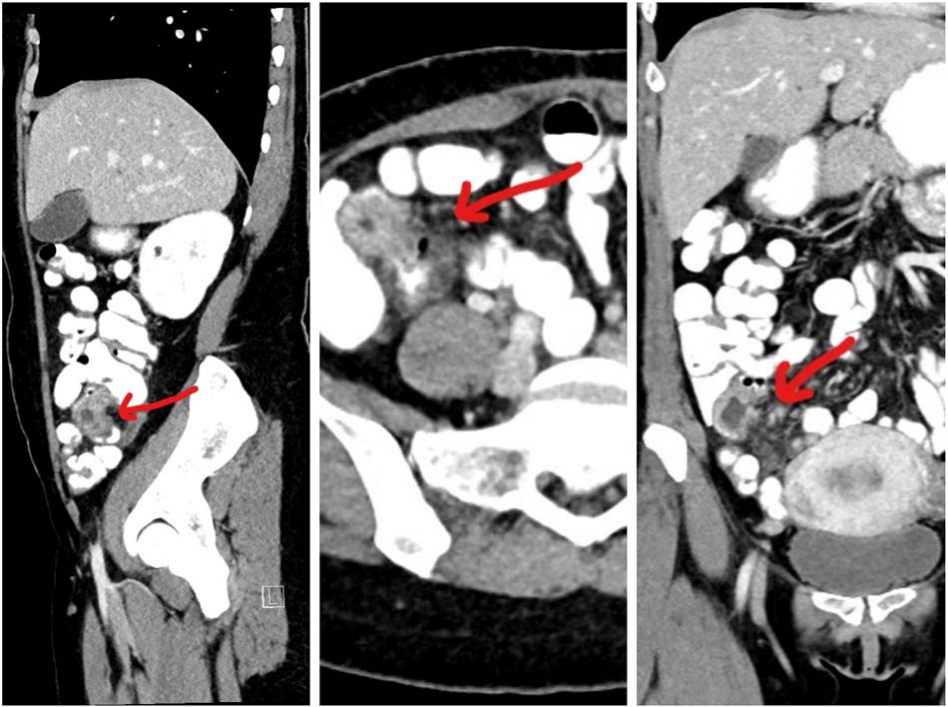
CT images showing the lesion in the right iliac fossa.

**Figure 2:**
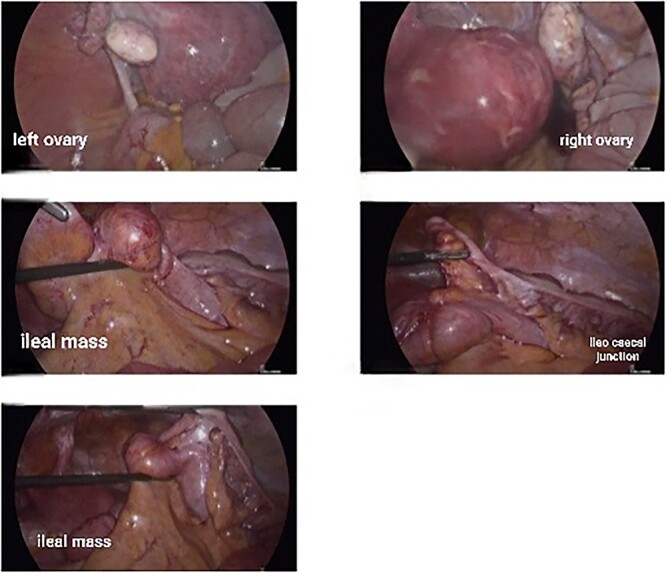
Intraoperative laparoscopic pictures showing normal uterus, ovaries, and the ileal lesion.

**Figure 3:**
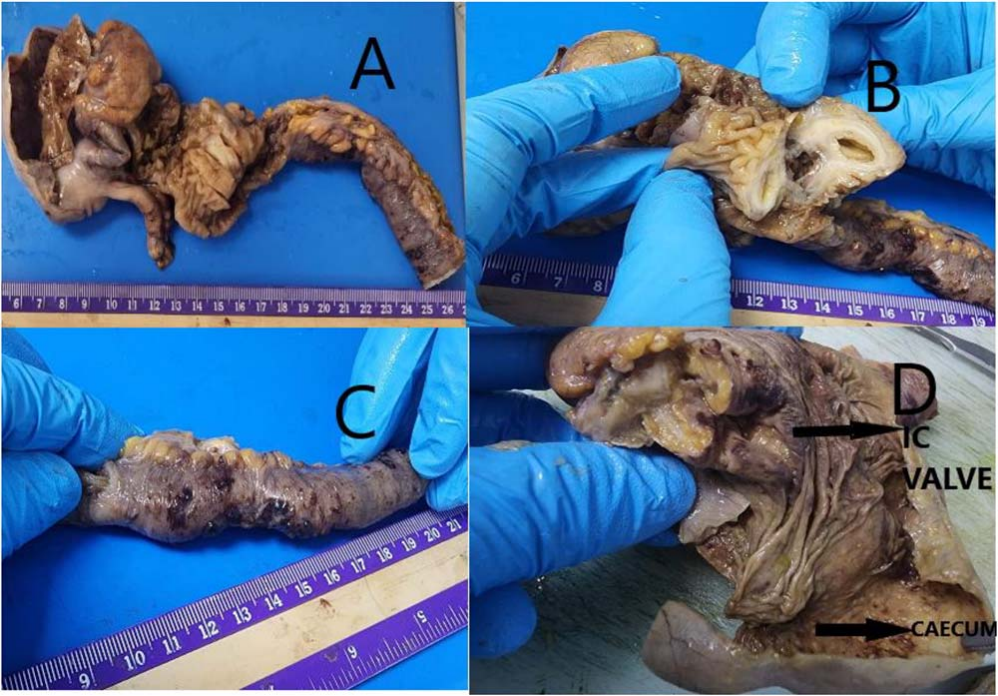
(A) Received partially cut opened limited hemicolectomy specimen. Ileum measuring 17 cm in length, 2 cm in diameter. Caecum dilated measuring 7.5 × 4.5 × 0.5 cm. Appendix identified measuring 4.0 × 0.4 cm. (B) Ileum shows already cut opened mass measuring 2.5 × 2.0 × 1.0 cm. Protruding into lumen. Cut surface showing cystic change with mucoid material. (C) Ileal end shows stricture for length 5 cm with serosa showing reddish hemorrhagic spots. (D) Ileocaecal valve shows congestion with brownish area. Caecum dilated wall thinned out with loss of mucosal rugosity. Representative sections submitted.

This 35-year-old nulliparous woman has been suffering from a long-standing undiagnosed abdominal pain that started 2 years earlier. The pain was colicky, localized in the right iliac fossa and seldom associated with nausea and vomiting. NO association with menstrual cycle. The previous episode was 3 months back when she was diagnosed at a center outside as a case of appendicitis with appendicular mass formation. The patient was treated conservatively at that time. This visit to the ER she presented with severe abdominal pain and subacute intestinal obstruction. The laboratories evaluations were normal. Patient had abdominal pain, nausea, vomiting, and constipation, but passing flatus for 2 days. She was vitally stable. Her abdomen was mildly distended with tenderness over the right lower quadrant but no signs of peritonitis. Patient was resuscitated and a CT scan with contrast was done to determine the nature of the obstruction (Fig. 1).CT scan showed a complex cystic lesion in the ileo caecal area with no proper delineation of the appendix. A clinical diagnosis of appendicular mass was made based on the CT findings and the patient was treated conservatively. But the patient did not respond well to conservative management and had persistent pain. She was planned for surgical intervention with a diagnostic laparoscopy and proceeded. A mass involving about 4 cm of the terminal ileum was found just about 4 inches away from the ileo caecal junction (Fig. 2). The serosa of the ileum looked normal. Appendix was found to be normal. Proximal to this intestinal lesion, there was another ileal stricture about 6 inches proximal to the ileal mass. Remaining part of the ileum and jejunum looked normal. The uterus, both ovaries, and fallopian tubes were normal (Fig. 2). Giving its close proximity to the ileocecal valve, decision was made to proceed with laparoscopic resection of the ileum including both the lesions and a limited resection of the ascending colon (Fig. 3) as discussed previously with patient.

**Figure 4:**
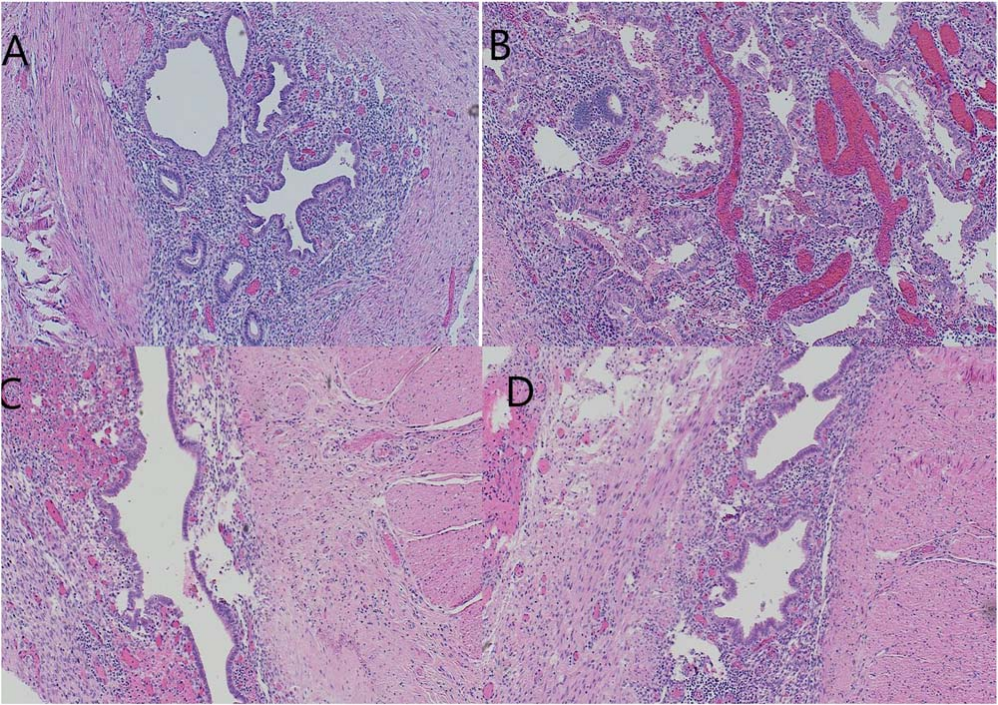
(A, B) Ileum with cystic structure showing submucosal endometrial glands and stroma plus hemosiderin extending to deeper layers of intestine surrounded by smooth muscle layer. (B) Ovarian tissue with diffuse areas of hemorrhage and necrosis. (C, D) Multiple mural and serosal foci of endometriosis noted corresponding to area of ileal stricture. Final diagnosis given as ILEUM WITH ENDOMETRIOMA WITH MULTIPLE MURAL AND SEROSAL FOCI OF ENDOMETRIOSIS. NEGATIVE FOR MALIGNANCY. MARGINS OF RESECTION UNREMARKABLE. TWO BENIGN LYMPH NODES.

Biopsy report came as invasive endometrioma of the ileum involving the muscularis layer and also additional stricture of the ileum. There was an involvement of the ileocecal junction as well (Fig. 4).

## Discussion

Endometriosis is characterized by the presence of functional endometrial tissue consisting of glands and stroma outside the uterus. Intestinal obstruction could be the presentation of ilial involvement. However, this is very rare up to 23% of all cases with ileum involvement [1]. Sampson’s retrograde menstruation theory is the most widely accepted theory. Endometrial tissue refluxes through the fallopian tubes, implanting on the serosal surface of abdominal and pelvic organs which commonly occurs during menstruation [5]. However, other theories and factors, immunological, genetic, and familial, could be involved in the pathogenesis of this disease [6]. Endometriosis presents usually with pelvic pain, infertility, and dyspareunia [7], but it may often be nonspecific. Because of terminal ileum involvement, our patient had recurrent pelvic pain with associated nausea and vomiting as a picture of partial small bowel obstruction.

Our patient is not married and her symptoms were not associated with menses and relapsed irregularly. Many GI diseases including small bowel obstruction, inflammatory bowel disease, and neoplasm make preoperative diagnosis more elusive because of clinical similarities [7]. Relapsing symptoms are the hallmark of this disease [7]. Endometriosis of the distal ileum is an infrequent cause of intestinal obstruction, ranging from 7% to 23% of all cases with intestinal involvement [8]. The incidence of intestinal resection for bowel obstruction is 0.7% among patients undergone surgical treatment for abdomino-pelvic endometriosis [9]. However, endometriosis of the small bowel should be suspected in young, nulliparous patients with abdominal pain, in conjunction with signs of obstruction as this is the case in our patient [3].

Imaging work up may be inconclusive. Endoscopy and barium enema are not helpful because the disease does not involve the mucosa. CT scan and MRI may be helpful in diagnosis of the condition [10]. Diagnostic laparoscopy is considered gold standard. The diagnosis can be confirmed only on histology. Gastrointestinal endometriosis is usually found as an incidental finding on abdominal exploration. Asymptomatic and mildly symptomatic cases may be treated using hormonal treatment.

Suspicion of malignancy as well as acute obstructive cases may warrant a radical resection [7]. The management should include hormonal therapy and surgery. The former treatment with danazol or gonadotrophin-releasing hormone analogs may be used in patients without obstruction. However, resection of the involved bowel remains the choice of treatment for complicated or unresolved cases [11]. In our case, our patient had a prolonged history of relapsing symptoms for years with picture of recurrent partial small bowel obstruction in addition to an image showing possible mass causing small bowel stenosis and obstruction. Because of those reasons, we elected to perform diagnostic laparoscopy and found this ileal and performed limited right hemicolectomy. The diagnosis was proven with histopathology (Fig. 3). Postoperatively, she has fully recovered with no abdominal complaints. She is on regular follow-up visits for assessment.

## Conclusion

Small bowel endometriosis is a rare entity. Clinical picture is similar to other gastrointestinal diseases and careful assessment is warranted. Surgical resection is sometimes indicated for diagnosis or failing medical management. High suspicious of index is warranted in these cases.
